# Longitudinal analysis of fecal tryptophan metabolites and microbiome composition in very preterm infants: impact of birth mode and feeding type

**DOI:** 10.1080/19490976.2025.2541031

**Published:** 2025-08-26

**Authors:** Naomi V. Wieser, Yannick van Schajik, Mohammed Ghiboub, Nina M. Frerichs, Roni Weiss, Mark Davids, Tim G. J. de Meij, Hendrik J. Niemarkt, Antoine Lefèvre, Patrick Emond, Joep P. M. Derikx, Wouter J. de Jonge, Bruno Sovran

**Affiliations:** a Tytgat Institute for Liver and Intestinal Research, Amsterdam University Medical Center, University of Amsterdam, Amsterdam, The Netherlands; b Department of Gastroenterology, Endocrinology, Metabolism (AGEM), Amsterdam University Medical Centers, Amsterdam, The Netherlands; c Department of Pediatric Surgery, Emma Children’s Hospital, Amsterdam University Medical Center, University of Amsterdam, Amsterdam, The Netherlands; d Amsterdam UMC, University of Amsterdam, Amsterdam Reproduction and Development Research Institute, Amsterdam, The Netherlands; e Department of Gastroenterology and Hepatology, Amsterdam University Medical Centers (UMC), University of Amsterdam, Amsterdam, Netherlands; f Department of Experimental Vascular Medicine, Amsterdam UMC, Amsterdam, The Netherlands; g Department of Pediatric Gastroenterology, Amsterdam University Medical Center, Amsterdam, The Netherlands; h Department of Neonatology, Maxima Medical Center, DB Veldhoven, The Netherlands; i Department of Electrical Engineering, Technical University Eindhoven, AE Eindhoven, The Netherlands; j UMR 1253, iBrain, University of Tours, Tours, France; k In Vitro Nuclear Medicine Laboratory, Regional University Hospital Center of Tours University, Tours, France; l Department of Surgery, University Hospital Bonn, Bonn, Germany; m Emma Center for Personalized Medicine, Amsterdam University Medical Center, University of Amsterdam, AZ Amsterdam, The Netherlands; n Toxalim (Research Centre in Food Toxicology), Université de Toulouse, INRAE, ENVT, INP-Purpan, UPS, Toulouse, France

**Keywords:** Preterm neonates, microbiota, tryptophan metabolism, cesarean-section, supplemented feeding

## Abstract

Preterm birth is associated with increased morbidity and mortality due to factors such as prolonged NICU stays, antibiotic treatments, and immature gastrointestinal and immune systems. These factors disrupt early gut microbial colonization, yet the functional metabolic implications remain understudied. Emerging evidence highlights the role of tryptophan metabolism as a mediator of host–microbiome interactions, critical for intestinal homeostasis and immune regulation. However, its dynamics and relationship with microbial composition in preterm infants are poorly understood. In this study, we longitudinally characterized the microbiome and 21 fecal bioactive tryptophan metabolites during the first month of life in 53 very preterm infants <30 weeks of gestation. Targeted tryptophan metabolomics and 16S rDNA sequencing revealed that cesarean delivery and supplemented feeding was associated with elevated host-derived kynurenine metabolites. Breastfeeding promoted beneficial microbiome colonization, including increased *Staphylococcus* and reduced *Proteobacteria*. Notably, *Bifidobacterium* abundance positively correlated with the AhR ligand indole-3-lactic acid, while *Staphylococcaceae* negatively associated with indole derivatives. Our findings underscore the link between diet, microbial composition, and tryptophan metabolism in very preterm infants. This work provides a foundation for exploring tryptophan’s role in development, health, and disease, emphasizing the importance of early nutritional strategies.

## Introduction

Preterm birth poses significant risks of mortality and morbidity despite improved survival rates in recent years.^1–3^ Preterm infants face metabolic challenges due to underdeveloped organ systems, including altered milk digestion caused by insufficient pancreatic function, immature gastrointestinal motility, and reduced intestinal nutrient absorption.^4–7^ These challenges highlight the urgent need for specialized nutritional strategies to support optimal development.

Additionally, factors such as cesarean section (c-section) delivery, antibiotic exposure, supplemented feeding and hospital care in neonatal intensive care units (NICU) disrupt early microbial colonization in preterm infants, leading to reduced levels of beneficial bacteria (e.g., *Bifidobacteriaceae*, *Lactobacilli*) and increased pathobionts (*Enterobacteriaceae*, *Enterococci*, *Staphylococci*).^8–11^ These shifts in microbiome composition are associated with an increased risk of severe conditions like necrotizing enterocolitis (NEC), bronchopulmonary dysplasia (BPD), and late-onset sepsis (LOS), while also potentially impacting nutrient metabolism, including that of essential amino acids like tryptophan.^12^

Tryptophan in the first month of life originates from milk and plays a central role in numerous physiological processes, serving as a precursor for protein synthesis and being integral to the biosynthesis of essential bioactive molecules, such as serotonin and melatonin^13^ . Previous studies in adults have shown that tryptophan metabolism is essential for cellular homeostasis and its dysregulation is related to various diseases.^14,15^ Particularly in the gut, a dysregulated tryptophan metabolism has been linked to inflammatory bowel disease (IBD), irritable bowel syndrome, and metabolic syndrome, and may present itself as a valuable therapeutic target.^16–18^

In early life research, maternal tryptophan metabolites from mothers’ milk, particularly indoles, have been shown to protect against NEC in a mouse model.^19^ The dynamics of tryptophan metabolism in preterm infants during the first month of life are a critical area of study with implications for growth, development, and overall well-being.^20,21^ Understanding these dynamics is particularly important given the unique challenges faced by preterm infants, including immature organ systems and altered gut microbiota. Therefore, it is essential to increase knowledge on the kinetics and pathways of tryptophan metabolism in preterm infants, focusing on how these are influenced by mode of birth and diet. These data could provide valuable insights into optimizing nutritional strategies and improving outcomes for preterm neonates.

We have previously demonstrated altered tryptophan metabolism in preterm compared to full-term neonates.^21^ We further showed that these altered metabolite concentrations affect chemokine production in a human fetal organoid model. While a few studies have highlighted the intricate interplay between tryptophan metabolism and the specific demands of preterm infants,^20,22^ our current study is among the first to assess the metabolism of tryptophan in preterm neonates longitudinally during the first month of life. Here, we employed a multi-omics approach, combining metabolomics data, from Liquid chromatography-Mass Spectrometry (LC-MS) and microbiome data (16S sequencing) from the fecal samples of preterm infants who did not develop complications, e.g. NEC and LOS, associated with microbiota/gut health to understand the metabolism of tryptophan in the first month of in these infants. This longitudinal multi-omics approach, investigating the effects of mode of delivery, diet, and its associations with the developing premature microbiome offers new insights into understanding tryptophan metabolism in preterm infants. Defining a baseline of tryptophan metabolism in this population will help providing a strong baseline to help elucidate potential associations of diet, delivery, and metabolism with health complications such as intestinal infections such as NEC and sepsis, immune development, and other critical aspects of early life physiology.

## Materials and method

### Preterm neonates and fecal samples

This study is part of a multicenter prospective cohort study across The Netherlands and Belgium (protocol 2014.386, amendment A2016.363) that included all preterm infants born below 30 weeks of gestation with daily fecal and clinical data collection up to the age of 4 weeks.^23^ Samples were collected from three centers: Isala Hospital Zwolle, Maastricht University Medical Center, and University Hospitals Leuven. Eligible infants did not develop LOS or NEC within the first 28 days of life. Sample collection, storage, and transportation were standardized across all centers. They were collected once a day by a nurse from birth through day 28 and placed in 10 mL polystyrene containers (Stuhlgefäß). Within an hour of collection, they were frozen at −20°C and then kept at −70°C. In case of transportation, samples were always transported on dry ice (−70°C). Sampling stopped if the infant was transferred to another hospital or passed away.

Clinical data, such as occurrence of comorbidities, antibiotic administration, follow-up duration, and type of feeding were gathered from the patients’ files (Table 1 , Supplementary Figures S1 and S2). None of the participating centers routinely provided probiotics or donor milk.^24,25^ Parenteral nutrition (PN) was administered according to protocol, starting right after birth, and was complemented with enteral feeds until the infant had reached full enteral feeding. Following achievement of full enteral feeds, PN could be resumed during any subsequent nil per os periods (Supplementary Figure S3 (a,b)).

**Table 1.** Patient characteristics.

Characteristics	MOM/Vaginal (*n = 12*)	MOM/ C-section (*n = 15*)	Supplemented/ Vaginal (*n = 8*)	Supplemented/ C-section (*n = 18*)
Male (%)	58	67	57	83
Gestational Age (weeks+days)	26 + 4 weeks ±11 days	27 + 4 weeks ±10 days	28 + 1 weeks ±10 days	28 + 4 weeks ±7 days
Birth Weight (gr)	1027.3 ± 250.4	982.8 ± 318.4	1237.4 ± 277.7	1110.778 ± 250.1
Average Days Antibiotic use in the first 28 days Antibiotic use in the first 28 days (Yes, %)	7.0 100	5.64 100	7.5 100	5.83 88.9
Total days follow up	26 ± 4.4	27.1 ± 3.4	25.8 ± 2.7	28.5 ± 0.5
Average number of days on parenteral nutrition (days)	13	9.5	9.4	12.7
Day of life reaching full enteral feeding (days)	13.08 ± 4.67	11.28 ± 2.43	12 ± 4	12.71 ± 3.47
APGAR Score 5 min (median [IQR])	8 [8–9]	7 [6–8]	7 [7–8]	8 [7–9]
Parity (%): 1 2 3	90.9% 9,1% 0%	71.4% 28.6% 0%	86.7% 6.7% 6.7%	27.8% 61.1% 11.1%

The infants were categorized into subgroups based on mode of birth (C-section versus vaginal delivery), followed by feeding type. Feeding type was defined as follows. First, the central feeding type (either exclusive mothers’ own milk (MOM), exclusive formula feeding, or mixed (MOM and formula feeding) was defined per day for the total follow-up duration. This included days where PN was supplemented with enteral feeds; however, days where only PN was administered were excluded from the calculation. The mothers’ own milk (MOM) group was defined as infants who received exclusively MOM for >30% of the enteral feeding days during the follow-up duration. The supplemented milk group were the infants who received either exclusively formula milk, or MOM with supplementation of formula milk, for more than 30% of the enteral feeding days during follow-up Supplementary Figure S2 (a-f). According to the protocols of the participating centers, infants in both groups could receive fortifiers if deemed necessary by the treating pediatricians.

**Figure 1.**
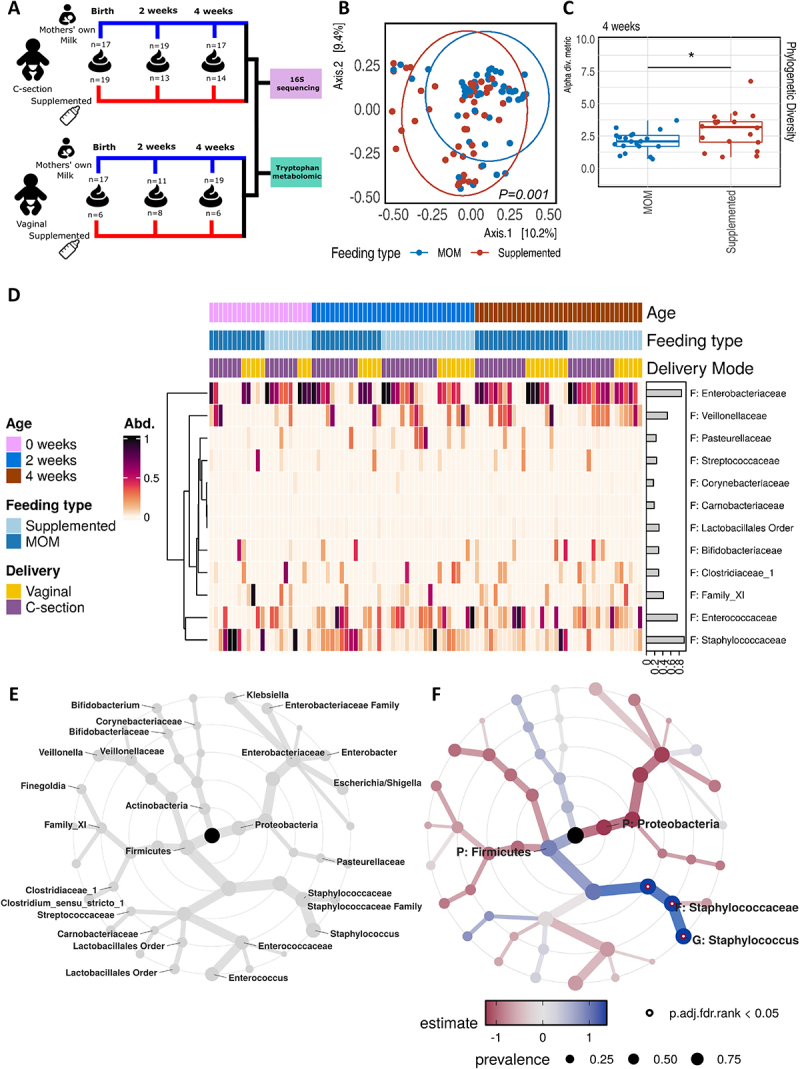
Microbial colonization of extremely and very preterm neonates in the first 4 weeks of life.

To assess the longitudinal microbiota composition and tryptophan metabolism, fecal samples from these infants were selected at different time points: at birth (*n* = 49), 2 weeks (*n* = 51) and 4 weeks (*n* = 48) postnatal age. From the 53 infants, 44 provided samples for all three time points, 7 provided for two time points, and 2 provided only one sample Figure 1(a).

(A) Overview of study subjects and samples collection: A total of 166 fecal samples were longitudinally collected and analyzed from 53 included patients included in this study. (B) Total beta diversity, visualized by Bray–Curtis distance, indicates significant clustering based on feeding type (PERMANOVA p = 0.001 at 999 permutations). (C) Faith’s Phylogenetic Diversity between MOM and supplemented milk fed groups at 4 weeks of life. (D) Heatmap illustrating the relative microbial abundance at family level, clustered by age, feeding type and delivery mode. The prevalence across all samples is shown in histogram at the bottom of the plot. (E) Taxonomic tree plot: nodes represent taxonomic levels from kingdom in the center to family at the edge. (F) Tree plot of microbiota associated with MOM feeding. Hollow dot represents FDR. Adj. *p* value <0.1, with dot size represents prevalence. P-values = *<0.5, **<0.01, ***<0.001, ****<0.0001.

See Supplementary Figure S1-3 and Table 1 for detailed information per infant. Antibiotic administration protocols were predominantly broad-spectrum and of comparable duration across feeding groups (Supplementary Figure S4(a)).

### Tryptophan metabolomics

Preterm neonatal fecal samples were processed to measure tryptophan metabolites using Liquid Chromatography-tandem Mass Spectrometry (LC-MS) coupled with triple quadrupole mass spectrometry (LC-MS/MS). The concentrations of tryptophan metabolites in fecal samples were quantified as previously described.^26^ In summary, sample preparation involved the following: 3 mg of lyophilized fecal material was mixed with 900 μL of a 1:1 methanol/water mixture along with internal standards. LC-MS/MS analysis was conducted as previously reported. Calibration curves were generated for each metabolite by calculating the intensity ratios between the metabolites and their respective internal standards. The concentrations of each metabolite in the fecal samples were determined using these curves. Specific calibration ranges, internal standard concentrations, and fragmentation parameters have been detailed in earlier research.^27^

Tryptophan metabolites in kynurenine, serotonin, and indole metabolism pathways were quantified. The kynurenine pathway metabolites include picolinic acid, 3-hydroxykynurenine, quinolinic acid, kynurenine, 3-hydroxyanthranilic acid, kynurenic acid, and xanthurenic acid. The serotonin pathway metabolites include 5-hydroxytryptophan, N-acetyl-serotonin, 5-hydroxyindoleacetic acid, and serotonin. The indole pathway metabolites include tryptamine, indole-3-acetamide, indole-3-lactic acid, indole-3-aldehyde, indole-3-acetic acid, tryptophol, and indole-3-sulfate.^28^ Tryptophan metabolism was evaluated by quantifying the total metabolites involved in each pathway.

### Bacterial DNA isolation

Bacterial DNA was isolated from frozen preterm neonate fecal samples (Stored at −80°C) following the PSP Spin Stool DNA Plus Kit protocol (Invitek Molecular/Isogen, Utrecht, The Netherlands) as described previously.^29^ Briefly, the fecal samples and negative controls were homogenized in Stool DNA Stabilizing buffer using a FastPrep bead beater. The samples were then warmed up to 95°C for 15 min and subsequently kept on ice for 1 min. Following this, samples were transferred to PSP InviAdsorb tubes, and manufacturer’s protocol was followed from there on. Total DNA concentration was measured using Nanodrop 1000 microvolume spectrophotometer (Thermo Fisher Scientific).

### Bacterial profiling of fecal preterm samples

Generation of 16S rRNA gene amplicons was performed using a single-step PCR procedure targeting the V3–V4 region, carried out at the Microbiota Center Amsterdam (MiCA) as described earlier.^30^ A minimum abundance of 4 reads was used to infer amplified sequence variants (ASVs) for each sample individually. Unfiltered reads were mapped against the collective ASV set to establish the abundances.^31^ Taxonomy was assigned using the IDTaxa^32^ and SILVA 16S ribosomal database V13.^33^ Data handling was performed using tidyverse.^34^ Alpha diversity was assessed by observed species, Shannon diversity, and Faith’s phylogenetic diversity using Phyloseq (v.1.48.0) and ggpubr (v.0.6.0).^35,36^ Alluvial plots were generated with the ggalluvial package (v.0.12.5).^37^ Beta diversity was assessed by Bray–Curtis distance, PERMANOVA was performed with adonis2 function from the vegan package (v.2.6–6.1) and adjusted to covariables based on the following formula: distance_matrix ~ Feeding +Time_Point + Delivery + Birthweight + Gestational_age with marginal effects.^38^ Heatmaps were plotted using MicroViz2 (v.0.12.3) package and ggplot2 (v.3.5.1).^34,39^ Spearman correlation and resulting heatmaps as well as taxonomic tree plots were generated using MicroViz2 (v.0.12.3).^39^ The correlation of relative abundance to feeding type was assessed by MAASLIN2 (v.1.18.0).^40^ The correlations of log2 transformed relative abundance and tryptophan metabolite concentration were plotted using ggplot2 (v.3.5.1) and ggpmisc (v0.5.6).^34,41^

### Statistical analysis

GraphPad Prism (San Diego, USA, v.9.1.0; v.10.2.0) was used for statistical analyses and graphical figures. Normal distribution of data was assessed using the Anderson-Darling, d’Agostino & Pearson, Shapiro-Wilk, and Kolmogorov-Smirnov. For two-group comparisons, either the nonparametric Mann–Whitney test or the two-tailed Student’s t-test was used, depending on data normality. Data are presented as means ± standard error of the mean (SEM) or median (interquartile range) from one of the two independent experiments. For multiple group comparisons, one-way analysis of variance (ANOVA) followed by Tukey’s post hoc test was used for normally distributed data, while the nonparametric Kruskal–Wallis test followed by Dunn’s post hoc test was applied for non-normally distributed data. Correlation analysis was conducted using pairwise comparisons of selected variables, with the following measures applied based on data type: Pearson correlation were computed using ‘stats 4.4.0’ for continuous numeric variables, Cramer’s v was computed using ‘vcd 1.4.13’ for categorical variables, and Point-Biserial Correlation were computed using ‘stats 4.4.0’ for combinations of numeric and categorical variables. To assess the relationship between metabolite levels and clinical or demographic metadata while handling multicollinearity and variable selection, we applied Elastic Net regression (Lasso and Ridge) using ‘glmnet 4.1.8.’ Prior to modeling, metabolite concentrations were log-transformed and standardized (Z-score) to ensure comparability across features. Statistical significance was set at *p* < 0.05. Specific statistical tests used are described in each figure legend.

## Results

### Microbiota colonization of extremely preterm neonates is mainly driven by diet and mode of birth

A cohort overview and fecal sample collection time points delineating patient stratification by delivery mode and dietary grouping, with a mean clinical follow-up duration of 27.30 (95% CI ± 0.97) days, are presented in Figure 1(a) and Supplementary Figure S1(a, b). Correlation analysis displayed low associations between baseline characteristics. Birthweight and gestational age were positively correlated with each other. Formula feeding was positively associated with both birthweight and gestational age, likely reflecting clinical practices in which infants with lower birthweight or gestational age are more frequently given formula. Feeding mode also correlated as expected: positively with infants who received formula and negatively with those exclusively receiving MOM with and without fortifiers (Supplementary Figure S1(k)). Duration of treatment and type of antibiotics did not significantly differ between patient groups (Supplementary Figure S4(a,b)). Subgroup analysis of antibiotic regimen revealed slightly prolonged amoxicillin administration in the MOM group, with extended vancomycin treatment specifically in the supplemented feeding group (Supplementary Figure S4(b)). Longitudinal assessment of microbial colonization by 16S rDNA gene sequencing-based microbial community analysis revealed that the timing of fecal sampling did not significantly affect beta diversity, as assessed by Bray–Curtis distance across the study population (Supplementary Figure S5(a)). In contrast, both the mode of birth (Supplementary Figure S4(b)) and feeding group (Figure 1(b)) exhibited significant differences in beta diversity. While no significant difference in beta diversity by mode of birth was observed at each specific time point, grouping by feeding group showed significantly different beta diversity at 2 weeks  (Supplementary Figure S5C). Alpha diversity metrics did not significantly differ between different birth modes as well as feeding type, except for Faith’s phylogenetic diversity at 4 weeks of age, which was higher in the supplemented feeding group (Figure 1(c), Supplementary Figure S6(a,b)).

Analysis of fecal samples collected immediately after birth revealed that MOM group was associated with a significant increase of *Firmicutes* at phylum level, whereas *Proteobacteria* were the most abundant phylum in the supplemented feeding group (Figure 1(d) and Supplementary Figure S6(c)) . During the first 4 weeks of life, the development of the microbiota in the MOM group showed a trend of decreasing *Firmicutes*, although no significant differences in relative abundance at phylum level were observed at 2 and 4 weeks after birth (Supplementary Figure S6(c)). Linear regression modeling on log2-transformed relative abundance data indicated trends of positive correlation between MOM feeding and *Staphylococcus* abundance and negative correlation with *Proteobacteria* abundance, compared to supplemented diet. These relationships are represented by a taxonomic tree plot, illustrating hierarchical levels from phylum (center) to genera (edges) (Figure 1(e,f)).

### Diet and delivery mode influence kynurenine pathway metabolites in fecal samples of preterm neonates

**Figure 2.**
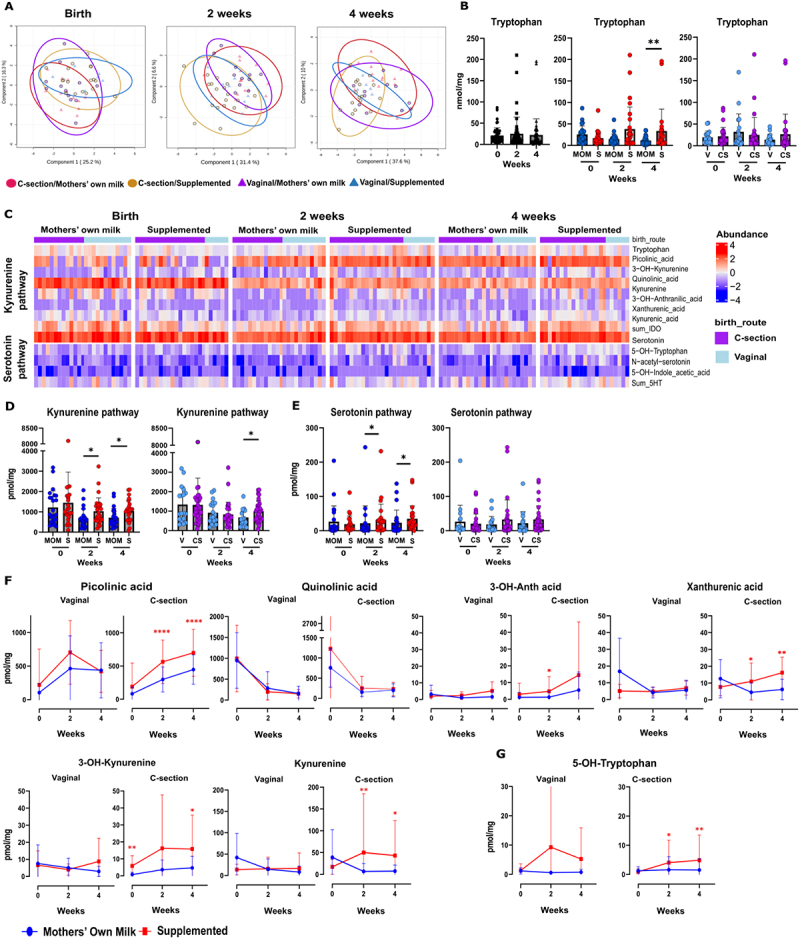
Supplemented milk fed and C-section delivered infants have higher levels of fecal kynurenine metabolites.

Since the gut microbiota composition is altered in preterm neonates, we aimed to better understand how preterm birth affects the metabolism of essential amino acids such as tryptophan. Here, we used a validated approach to quantify 21 tryptophan metabolites in the feces of preterm infants.^26^ First, we explored the effects of diet and delivery mode on the fecal tryptophan metabolome over the first month of life using sPLS-DA and an elastic net analysis (Figure 2(a), Supplementary Figure S7(c)). At birth, all four groups (categorized by diet and delivery mode) showed some overlap, although a slight clustering was observed among neonates in the MOM group. This trend became more pronounced over time, with a significant separation based on diet by 4 weeks (Figure 2(a) and Supplementary Figure S7(a-c)). This prompted us to investigate the effects of diet and delivery on the host metabolism of tryptophan, the kynurenine, and serotonin pathways. At 4 weeks, fecal samples from supplemented milk group neonates contained higher levels of tryptophan, suggesting alteration in the metabolism of tryptophan in this group (Figure 2(b)). We, therefore, investigated the metabolism of tryptophan by the host, namely the kynurenine and serotonin pathways (Figure 2(c)). At 2 and 4 weeks after birth, the fecal samples of supplemented feeding group preterm neonates showed significantly higher levels of metabolites from the sum of the kynurenine pathway (Figure 2(d)) (*p* < 0.05, ANOVA). Additionally, neonates born via C-section displayed higher levels of kynurenine metabolites at 4 weeks (*p* < 0.05, ANOVA). Notably, picolinic acid significantly increased over time, whereas quinolinic acid significantly decreased after birth (Figure 2(c)). When investigating the combined effects of diet and delivery on the metabolites, picolinic acid was significantly higher after 2 weeks in the C-section delivered supplemented fed group (Figure 2(f)). Similarly, 3-OH-anthranilic acid, xanthurenic acid, 3-OH-kynurenine and kynurenine were all significantly increased over time in fecal samples from C-section delivered supplemented milk group (Figure 2(f)). Next, we assessed the effect of diet and delivery on the metabolites of the serotonin pathway. The sum of the serotonin pathway metabolites displayed significantly higher levels of serotonin metabolites in the feces of the supplemented milk group neonates (Figure 2(e)). When combining diet and delivery, C-section and supplemented milk group fecal samples had a significant increase in the serotonin precursor 5-OH-tryptophan (Figure 2(g)). All other kynurenine and serotonin metabolites did not show significant differences between diet and delivery modes, although kynurenic acid displayed a similar increase trend in the feces of the infants born through C-section and supplemented milk fed group (Supplementary Figure S8(a,b)).

(A) sPLS-DA 2-dimensional plot of fecal metabolome of tryptophan at birth (*n* = 49), 2 weeks (*n* = 51) and 4 weeks (*n* = 48) after birth. Red spheres represent metabolites from C-section delivered and MOM fed neonates’ samples; brown spheres represent metabolites from C-section delivered and supplemented milk fed neonates’ samples, purple sphere represents vaginally delivered and MOM fed neonates’ samples and blue spheres represents vaginally and supplemented milk fed neonates’ samples. (B) Bar plots of fecal tryptophan concentration over time, divided by diet or divided by delivery mode. (C) Heatmap visualization describing the kynurenine and serotonin metabolites abundance overtime. Categories are divided by time points (birth, 2 weeks, and 4 weeks), diet (MOM *n =* 26 vs supplemented milk *n =* 27) and delivery mode (C-section *n =* 33 vs vaginal *n =* 20). (D) Bar plots of the SUM metabolites of the kynurenine pathway grouped by diet or delivery. (E) Bar plots of the SUM metabolites of the serotonin pathway divided by diet or delivery. (F) Line graph of the SUM metabolites of the kynurenine pathway divided by diet or delivery mode. (G) Line graph of the serotonin pathway metabolites divided based on delivery and diet. P-values = * <0.5, ** <0.01, *** <0.001, **** <0.0001. Normality check was performed. Data that passed the normality check were analyzed using student’s t-test; data that did not pass were analyzed using the Wilcoxon rank sum test was performed.

### Microbiota-derived indole is elevated in the fecal samples of C-section supplemented milk fed group preterm neonates

**Figure 3.**
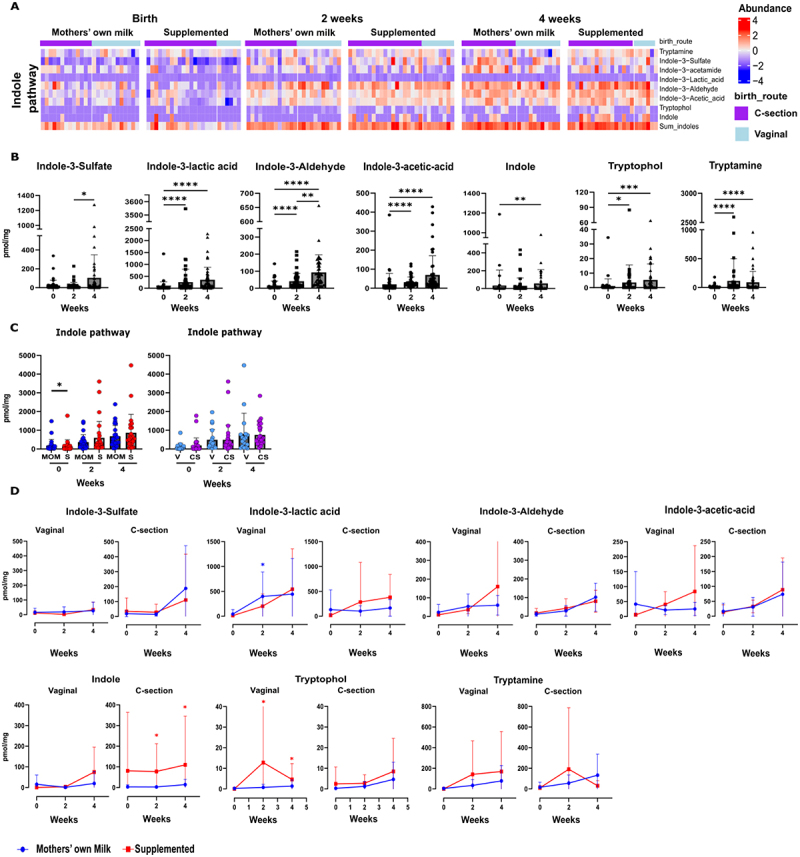
Elevated fecal indole levels in supplemented milk fed and C-section delivered preterm infants.

In a previous study, we have identified differences in the metabolism of tryptophan when comparing full-term and preterm infants.^21^ Furthermore, previous studies, as well as our cohort, have shown that the microbiome of preterm neonates is significantly altered by diet and delivery mode.^8,9^ To better understand the functional implications of preterm microbiome alterations, we evaluated the indole pathway metabolites in the feces of these preterm neonates (Figure 3(a)). After birth, the concentration of each indole metabolite significantly increased over time (Figure 3(a,b)). Interestingly, the sum of the indole pathway metabolites did not show significant differences based on diet or delivery method, except at birth, where feces from MOM group neonates exhibited higher concentrations of indole metabolites (Figure 3(c)). However, when considering both diet and delivery together, indole metabolites were significantly increased in fecal samples from neonates delivered through C-section in supplemented milk group from 2 weeks onward (Figure 3(d)). Furthermore, neonates born vaginally in supplemented milk group displayed significantly higher levels of tryptophol from 2 weeks on (Figure 3(d)). Moreover, indole-3-lactic acid was significantly higher at 2 weeks in the vaginally delivered, MOM group neonates, although that difference was no longer significant by 4 weeks (Figure 3(d)).

(A) Heatmap visualizing changes in indole pathway metabolites over time, categorized by time points [birth (*n* = 49), 2 weeks (*n* = 51), and 4 weeks (*n* = 48)], diet type (MOM vs supplemented milk), and delivery mode (C-section vs vaginal). (B) Bar plots illustrating the metabolite concentrations of individual indole pathway overtime. (C) SUM of the metabolite concentrations in the indole pathway, stratified by diet or delivery. (D) Line graph tracking indole pathway metabolite levels, stratified by delivery mode and diet type. P-values = * <0.5, ** <0.01, *** <0.001, **** <0.0001. Data normality was assessed. The data what passed the normality check were analyzed using student’s t-test. If the data did not pass the normality check, Wilcoxon rank sum test was performed.

### Feeding impacts on development of microbial colonization and tryptophan metabolism despite antibiotics treatment after birth

In this diverse cohort, we observed significant correlations between microbial abundance and tryptophan metabolites across taxonomic levels. Most indoles in the fecal compartment are derived from microbial metabolism, which are strongly related to total tryptophan bioavailability and downstream host metabolism.^28^

**Figure 4.**
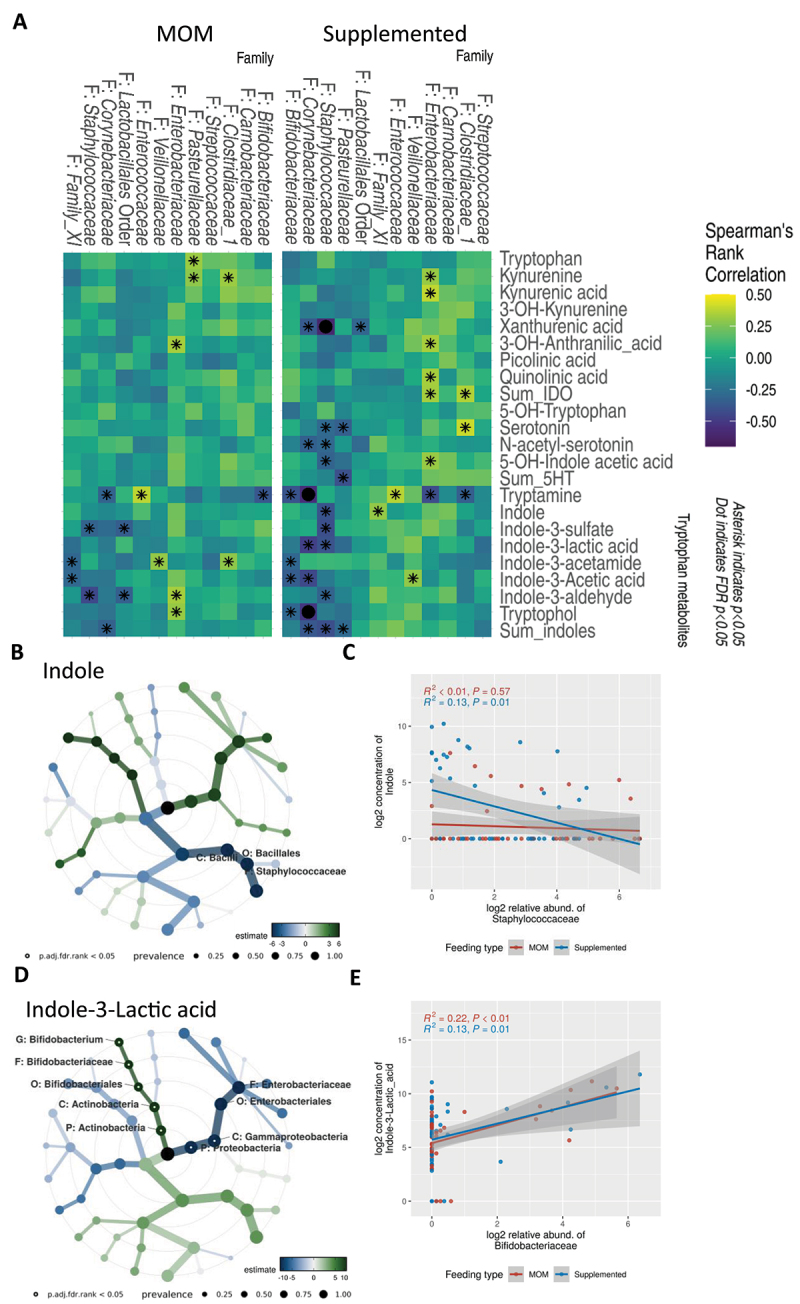
Colonizing microbiota are associated with tryptophan metabolism and influenced by diet.

**Figure 5.**
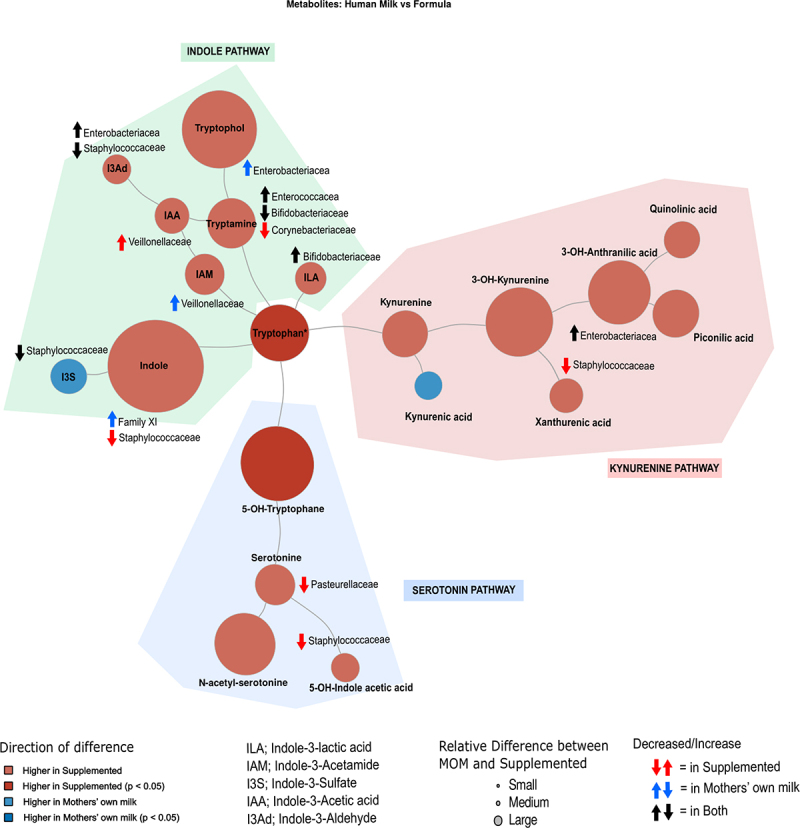
Overview of dynamics of tryptophan metabolites and microbial associations during the first 4 weeks of life.

To further investigate this, microbial to tryptophan metabolite associations were initially assessed by Spearman’s rank correlation, followed by linear modeling of log2 transformed taxonomic abundance at family level and log2 transformed metabolite concentrations. Across the study population, tryptophan metabolites predominantly exhibited a negative association with *Staphylococcaceae, Corynebacteriaceae*, and *Lactobacillales*, and a positive association with *Enterobacteriaceae, Enterococcaceae*, and *Bifidobacteriaceae* (Figures 4,5 and Supplementary Figure S9, Supplementary Figure Table S1). Other taxa showed weaker associations, partly due to their low abundance and prevalence (Figure 4(a)). Noteworthy, the supplemented milk group indicated a general shift in microbial associations with tryptophan metabolites, as an increased number of negative associations of microbial abundance and tryptophan metabolite levels were observed (Figure 4(a)). Observing microbial to tryptophan metabolite concentrations per sampling timepoint indicated a significant negative correlation of *Staphylococcaceae* and *Streptococcaceae* at 4 weeks of life (Supplementary Figure S8(a)). The abundance of tryptophan was associated to *Pasteurellaceae* in the MOM group, however linear modeling did not indicate a significant association (Figure 4(a)).

(A) Heatmap of Spearman’s rank correlation for each feeding group, comparing log2-transformed microbial taxa relative abundance at family level and log_2_-transformed tryptophan metabolite concentrations. *p* < 0.05 is marked with an asterisk, adjusted FDR p-values <0.05 are indicated with a dot. (B) Taxonomic tree plot showing associations between indole abundance and microbial taxa. Hollow dots represent FDR-adjusted p-values <0.1, with dot size corresponding to taxon prevalence. (C) Linear regression plot showing the relationship between log_2_-transformed concentrations of indole and log_2_-transformed relative abundance of *Staphylococcaceae*. (D) Taxonomic tree plot showing associations between indole-3-lactic acid abundance and microbial taxa. Hollow dot represents FDR-adjusted p-values < 0.1, with dot size indicating prevalence. (E) Linear regression of log_2_-transformed concentrations of indole-3-lactic acid and log_2_-relative abundance of *Bifidobacteriaceae.*

Node color represents direction of association of each metabolite with diet: red indicates an association with supplemented milk feeding, and blue with MOM, with darker shades denoting significant changes in metabolite levels. Node colors represent the direction of association of each metabolite with diet: Node size reflects the relative difference in metabolite levels between the MOM and supplemented milk groups. The tryptophan node is scaled down by a factor of 100 for visual clarity. Bacterial taxa names indicate significant association confirmed by both Spearman correlation and linear modeling of log_2_-transformed bacterial abundance against metabolite concentration. Black arrows indicate significant association in both supplemented milk and MOM groups, red arrows indicate association to supplemented milk group, blue arrows indicate association to MOM group. Upward arrows indicate positive association, while downward arrows indicate negative association. Abbreviations are indicated in the figure.

Within the serotonin pathway, distinct associations were observed in the supplemented milk group. Serotonin levels exhibited a significant negative correlation with *Pasteurellaceae* abundance (Figures 4(a), 5). Additionally, 5-OH-indoleacetic acid, a primary metabolite of serotonin, showed a significant negative association with *Staphylococcaceae* abundance (Figures 4(a), 5).

Kynurenine pathway metabolites are primarily host-derived; however, some significant associations with microbial abundance were observed.^42,43^ Specifically, the metabolites 3-OH-anthranilic acid and xanthurenic acid showed notable association with microbial abundance in this preterm infant cohort. 3-OH-anthranilic acid was positively correlated with *Enterobacteriaceae* levels in both diet groups (Figures 4(a), 5). Conversely, xanthurenic acid abundance was negatively associated to *Staphylococcaceae* levels (Figure 4(a), 5 and Supplementary Figure S9(b)).

Indole levels were positively correlated with Family XI *Clostridiaceae*, in the MOM group, but negatively correlated with *Staphylococcaceae* in the supplemented milk group (Figures 4(a-c) 5). Additionally, a slight trend of positive correlation to *Enterobacteriaceae* and *Veillonellaceae* was observed, independent of feeding mode (Figures 4(a), 5). The downstream metabolite of Indole, Indole-3-sulfate, was negatively associated with *Staphylococcaceae* in both groups (Figures 4(a), 5). Among other metabolites of the indole pathway, *Veillonellaceae* was positively associated to indole-3-acetamide in the MOM group, whereas in the supplemented milk group, *Veillonellaceae* was positively correlated with the metabolite of indole-3-acetamide, indole-3-acetic acid (Figures 4(a), 5). Indole-3-aldehyde, the downstream metabolite of indole-3-acetic acid, was negatively associated with *Staphylococcus* and positively associated with *Enterobacteriaceae* independent of diet (Figures 4(a), 5). Tryptamine, which can be produced by the intestinal microbiota through decarboxylation,^44^ was positively correlated with *Enterococcaceae* and negatively correlated with *Bifidobacteriaceae* in both groups (Figures 4(a), 5 and Supplementary Figure S9(c)). Notably, *Corynebacteriaceae* abundance was negatively correlated only in the supplemented diet group (Figures 4(a), 5). Tryptophol, the breakdown product of tryptamine, indicated a positive correlation to *Enterobacteriaceae* abundance in the MOM group (Figures 4(a), 5 and Supplementary Figure S9(d)).

Notably, the microbial tryptophan metabolite indole-3-lactic acid, which is often reported to be associated with positive health outcomes in early life,^19,20^ was significantly associated with *Bifidobacteriaceae* family abundance in both feeding groups, indicating a shift from tryptamine to indole-3-lactic acid in samples with increased *Bifidobacteriaceae* abundance (Figures 4(a,d,e), 5). A further general correlation of indole-3-lactic acid to the family *Bifidobacteriaceae* was observed regardless of grouping, as well as a negative correlation to *Gammaproteobacteria* (Figures 4(a,d,e), 5).

## Discussion

Approximately 10% of births are preterm, substantially impacting the health and development of these infants.^45,46^ The impact of preterm birth on nutrient metabolism, including the interplay between the host and its developing microbiome, remains underexplored. This study focused on mapping microbial composition and tryptophan metabolism in the first month of life, which has been recognized in the adult population and is an emerging area of interest in early life.^21,28,47^ Tryptophan metabolites modulate cell signaling and immune responses through receptors aryl hydrocarbon receptor (AhR), α7 nicotinic acetylcholine receptor (a7nAChR), multiple ionotropic glutamate receptors, and orphan G protein-coupled receptor 35 (GPR35).^21,28,48,49^ In addition, the microbiome of very preterm infants is known to be affected by mode of delivery, the extensive use of antibiotics, the time in the NICU and diet composition, potentially impairing the colonization of beneficial bacteria.^50,51^ In this study, delivery mode and diet significantly influenced microbiota composition and tryptophan metabolism of preterm born neonates during the first month of life, despite widespread antibiotic use. Regardless of the extensive antibiotic use in almost all included infants, both feeding type and delivery mode significantly impacted microbiome composition, particularly, at the beta diversity. Although no significant differences in beta diversity based on diet were observed at birth, significant differences emerged by 2 weeks of age as the microbiome began to establish. Across our entire cohort, the timing of sampling did not result in strong differences in beta diversity. In line with previous findings,^8^ this possibly indicates altered initial microbiome development in preterm neonates compared to full-term neonates, who typically display a stepwise assembly of microbial species, particularly with an increase in *Bifidobacterium spp*.^52–54^ Feeding pattern and delivery mode significantly influenced beta diversity in this cohort. Alpha diversity has been described as low at birth due to the progressive colonization of the preterm gut by relevant species.^8,55^ In this study, we observed in overall low alpha diversity, with the only significant difference observed in Faith’s phylogenetic diversity between diet groups at 4 weeks postnatal age, potentially suggesting a slightly lower alpha diversity in the MOM group samples. Furthermore, MOM group neonates exhibited significantly higher levels of *Firmicutes* at birth, whereas supplemented fed preterm infants were predominantly colonized by *Proteobacteria* in the first week of life. Longitudinally, *Proteobacteria* gradually increased in the MOM group neonates, resulting in a comparable abundance of *Proteobacteria* and *Firmicutes* in both groups by 4 weeks after delivery. MOM group was associated with higher levels of *Staphylococcus*, a facultative anaerobe previously implicated in primary colonization.^56,57^ Interestingly, regardless of subject groups, *Bifidobacterium* which is a key tryptophan metabolizer in early life strongly associated with health-associated microbial colonization, constituted a minor proportion of the microbiome in these infants, potentially resulting in detrimental alterations in the metabolism of tryptophan compared to a healthy term population.^58,59^ It is important to highlight that in contemporary hospital settings, it is increasingly common for infants to receive combinations of feeding types, reflecting a broader shift toward personalized feeding strategies.^60^ However, this practice constitutes a limitation in our study, as it may influence the interpretation of outcomes associated to exclusive formula feeding. Taken together, these results indicate feeding mode as the main driver in developing microbiota in this early preterm cohort, affirming previous findings, as well as indicating a considerable proportion of genera that are capable of metabolizing tryptophan.^55,57,61,62^

Diet also impacted the fecal tryptophan metabolome in preterm neonates. After 4 weeks of age, supplementation-fed infants exhibited higher fecal tryptophan levels, likely reflecting differential utilization of tryptophan by the host and microbiome or different tryptophan levels in diet. Tryptophan levels in human milk fluctuates based on circadian rhythm as well as over time postnatally,^59^ whereas formula contains fixed tryptophan concentrations.^21,60^ Differences between human milk and formula were described previously and accounted for in this study.^21^ However, the differences in tryptophan could stem from diet, the host or the absorption. Specific host-derived metabolites, including kynurenine and picolinic acid, were elevated in C-section-delivered, supplementation-fed neonates. Elevated kynurenine pathway metabolites suggests that tryptophan is being metabolized through specific immune-regulated pathways, which could be due to inflammation or microbial imbalance, although studies have also highlighted a possible role in immune tolerance as well.^63–68^ Xanthurenic acid levels negatively correlated with *Staphylococcus* in supplementation-fed neonates, while *Staphylococcus* abundance positively correlated with MOM-fed neonates, highlighting diet-associated microbial interactions. Serotonin pathway differences were driven by elevated 5-OH-tryptophan in supplementation-fed neonates, possibly reflecting altered absorption or conversion. Since serotonin regulates sleep and neurological function, proper 5-OH-tryptophan metabolism is critical in neonates.^63–65^ These findings support the significant influence of diet and delivery mode on host tryptophan metabolism and highlight the complex interplay between early-life factors, metabolism, and the gut microbiome in preterm infants.

Following initial bacterial colonization, the levels of microbial-derived metabolites exhibited a progressive increase over time, with significant increase of all indole metabolites and peaking at 4 weeks of age. Indole-3-lactic acid was notably higher at 2 weeks in vaginally delivered MOM fed neonates, correlating strongly with *Bifidobacterium* abundance and negatively correlating with *Enterobacteriaceae, Staphylococcaceae*, and *Corynebacteriaceae* families. Although strongly correlated to indole-3-lactic acid concentrations, a low relative abundance of *Bifidobacterium* was observed in this study, consistent with previous findings in very early preterm neonates.^59^ This is likely related to the reduced transmission from mother to neonate due to preterm delivery and reduced levels of indole-3-lactic acid in preterm neonates.^21,59^ These results affirm the potential of *Bifidobacterium* as probiotic and its effect may be functionally related to tryptophan metabolism.^69,70^

C-section-born, supplementation-fed preterm neonates exhibited increased indole levels compared to vaginally delivered MOM-fed preterm neonates. Indole, a metabolite linked to *E. coli, E. faecalis*, and *Klebsiella*, is noteworthy due to its role as a signaling molecule influencing bacterial drug resistance, biofilm formation, and virulence.^71,72^ Although indole can activate AhR and limit *E. coli* division, indole levels were below activation thresholds in our study.^72–75^ Members of *Clostridia cluster XI*, while incapable of producing indole, may induce its production in *E. coli*.^73^

Tryptophol, another metabolite from the indole pathway, was higher in vaginally delivered, supplementation-fed neonates by 2 weeks. While tryptophol’s role in neonates is unclear, it may influence immune responses or interact with the developing.^76,77^ These findings emphasize the need to investigate multi- kingdom interactions and other microbiome constituents, such as bacteriophages and mobile genetic elements.^55,78^

This study’s limitations include the absence of a control group without antibiotic treatment, the limited sample size, and the use of 16S rDNA sequencing, which lacks strain-level resolution. The lack of a control antibiotic-free group reflects the clinical reality of preterm infant care, where most receive antibiotics, but future studies are needed for deeper insights. While the small sample size limits statistical power, the findings remain robust and provide a critical foundation for further research. Finally, although 16S rDNA sequencing does not offer strain-level resolution, it still provides valuable insights into microbial diversity and metabolism at the community level, which is sufficient for understanding broader metabolic pathways in preterm infants. In this study, measurement of a selection of tryptophan metabolites was performed, this selection does not fully cover all potential metabolites, although it does provide absolute quantification compared to other intensity-based methods.^26^ Additionally, not all infants contributed samples at each time point, which may introduce bias, although no samples were pooled across time points. Despite these constraints, this study demonstrates the significant impact of feeding type and delivery mode on microbiome development and tryptophan metabolism in preterm neonates.

In conclusion, this study demonstrates the significant influences of feeding type and delivery mode on tryptophan metabolism and microbiome development within the first month of life in very preterm neonates. This is one of the first studies to longitudinally integrate the effects of premature birth, closely monitored feeding intake and birth mode on microbial colonization and metabolic composition in a multi-OMIC approach. Despite the relatively small sample size, this cohort of extremely preterm neonates, who were heavily treated with broad-spectrum antibiotics, indicated the development of the microbiome and was most strongly impacted by feeding type, confirming and expanding upon previous results.^57^ In line with recent findings, our work underscores the urgent need to understand the metabolism of milks and supplementation in early life. Mix-feeding strategies and personalized nutrition methods should be continuously improved to promote healthy microbial colonization and decrease risk of morbidities such as BPD, LOS, and NEC.^79^

Understanding tryptophan metabolism in early life could inform targeted nutritional strategies to improve health outcomes in this vulnerable population. Further investigation is required to fully understand the dynamics and effects of tryptophan metabolism in early life and its effect on microbial colonization and dynamics, as well as host immune-, barrier-, and neurological development.

## Supplementary Material

Supplemental Material

## Data Availability

Sequencing data has been uploaded to ENA repository PRJEB83875.
