# Supplementary material for: Longitudinal analysis of fecal tryptophan metabolites and microbiome composition in very preterm infants: impact of birth mode and feeding type
Source: Gut Microbes. 2025 Aug 26;17(1):2541031. doi: 10.1080/19490976.2025.2541031 (PMC12382476; doi:10.1080/19490976.2025.2541031)
Supplement: Supplemental Material [file KGMI_A_2541031_SM4781.pdf]

Early life development: how diet and mode of birth affect tryptophan metabolism Supplementary material

Naomi V. Wieser[1, #*], Yannick van Schajik[1, #*], Mohammed Ghiboub [1,2,3], Nina M. Frerichs[3,4], Roni Weiss[1,5], Mark Davids[4], Tim G. J. de Meij [2,5], Hendrik J. Niemarkt [6,7] Antoine Lefèvre [8], Patrick Emond [8,9] , Joep P. M. Derikx [3], Wouter J. de Jonge [1,2,10] and Bruno Sovran [1,3,11*]

Supplementary figure 1 – Cohort characteristics

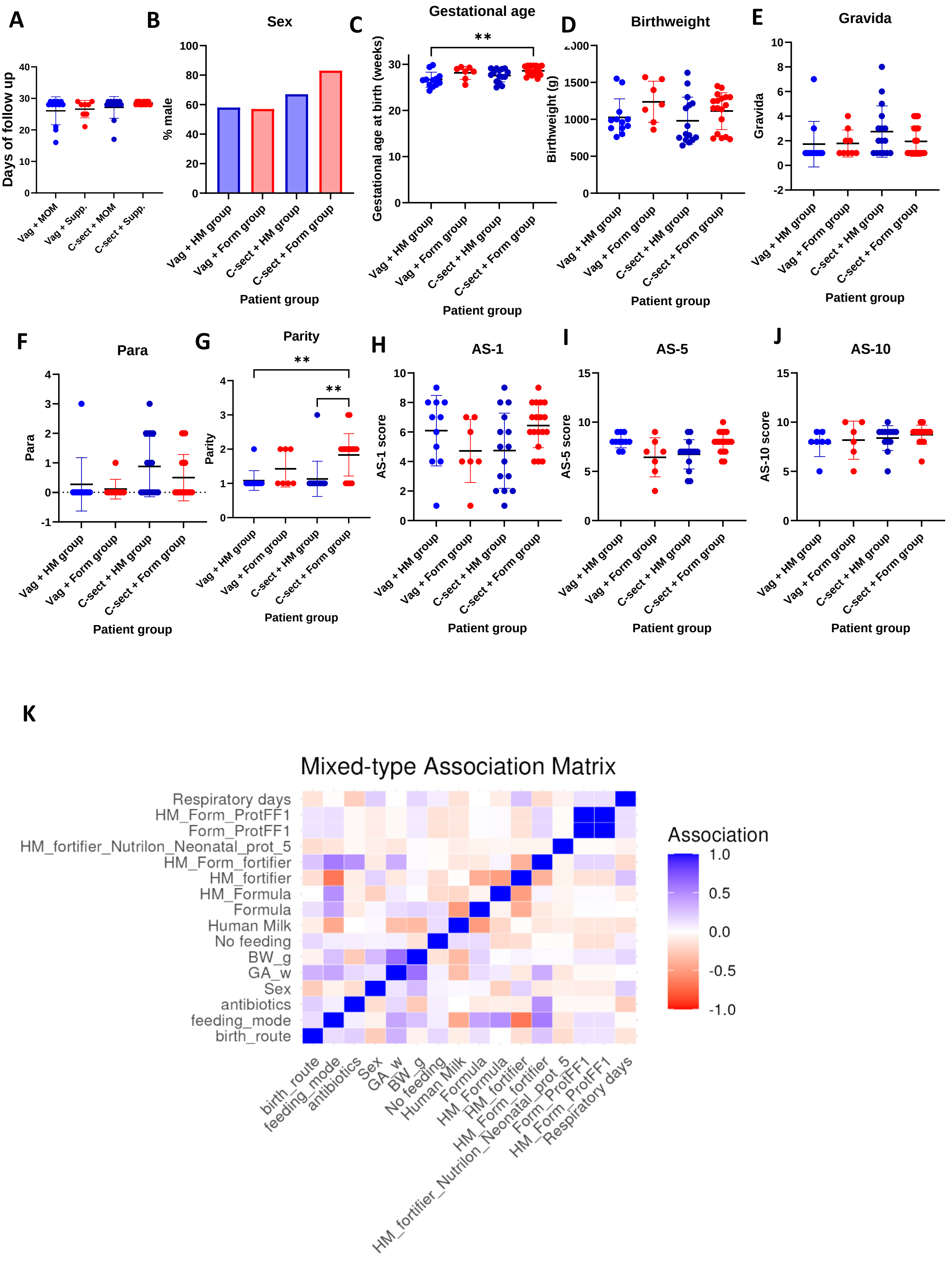

Supplementary figure 2 – Feeding strategy

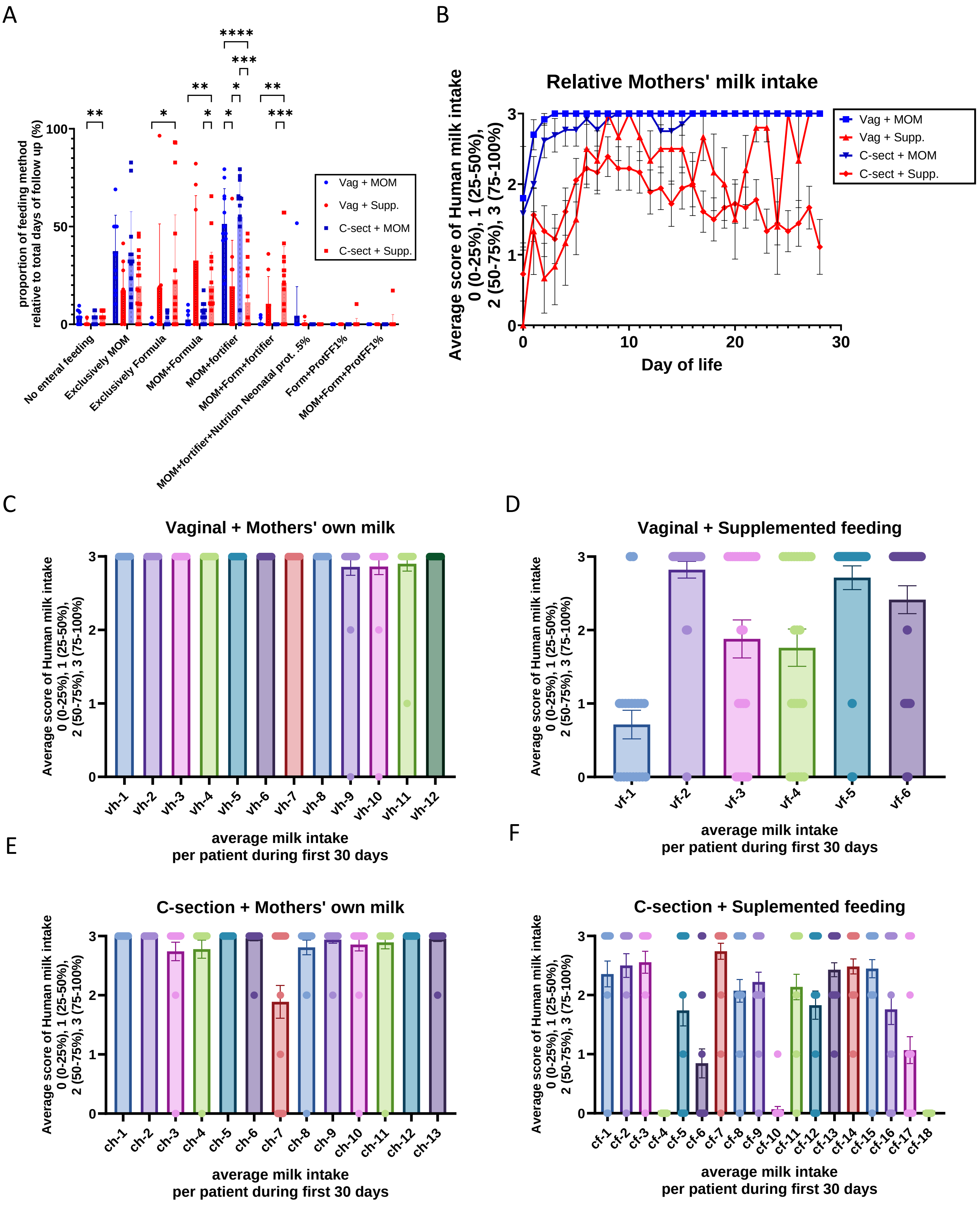

Supplementary figure 3 – Feeding quantity

A

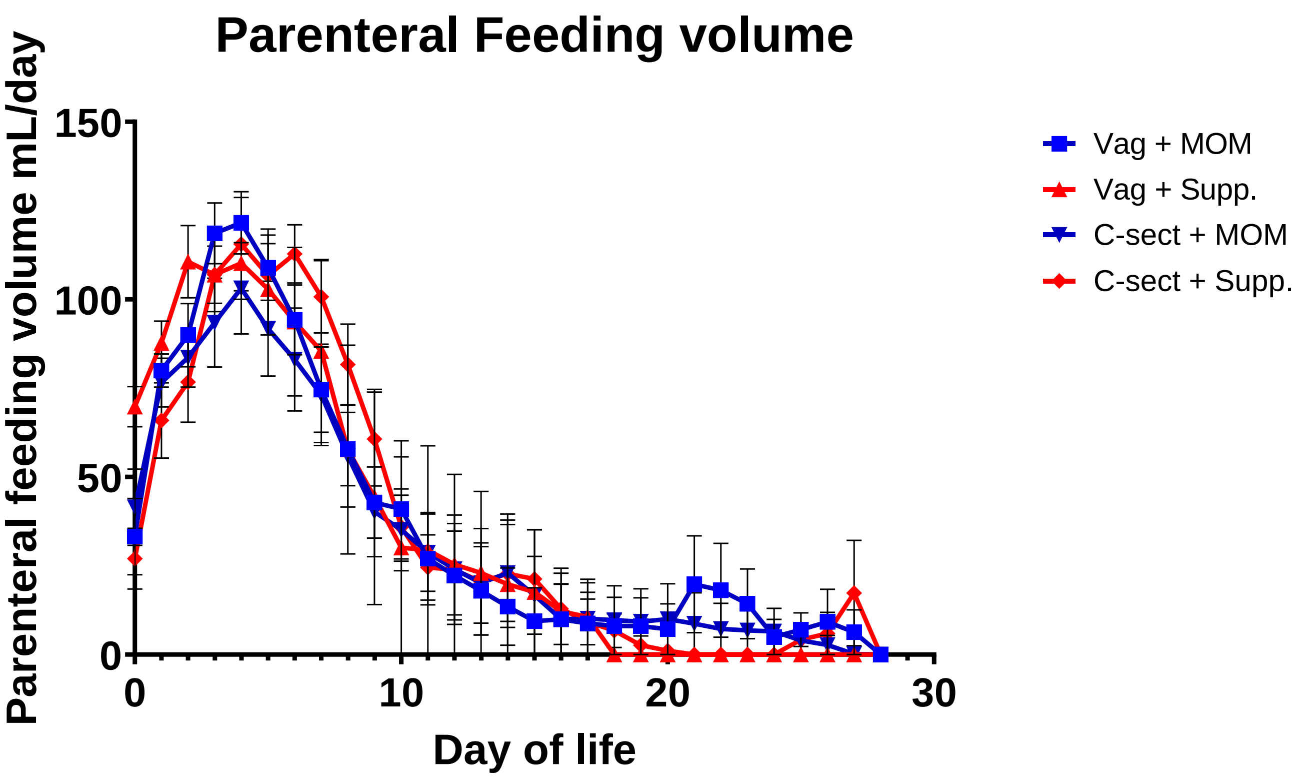

B

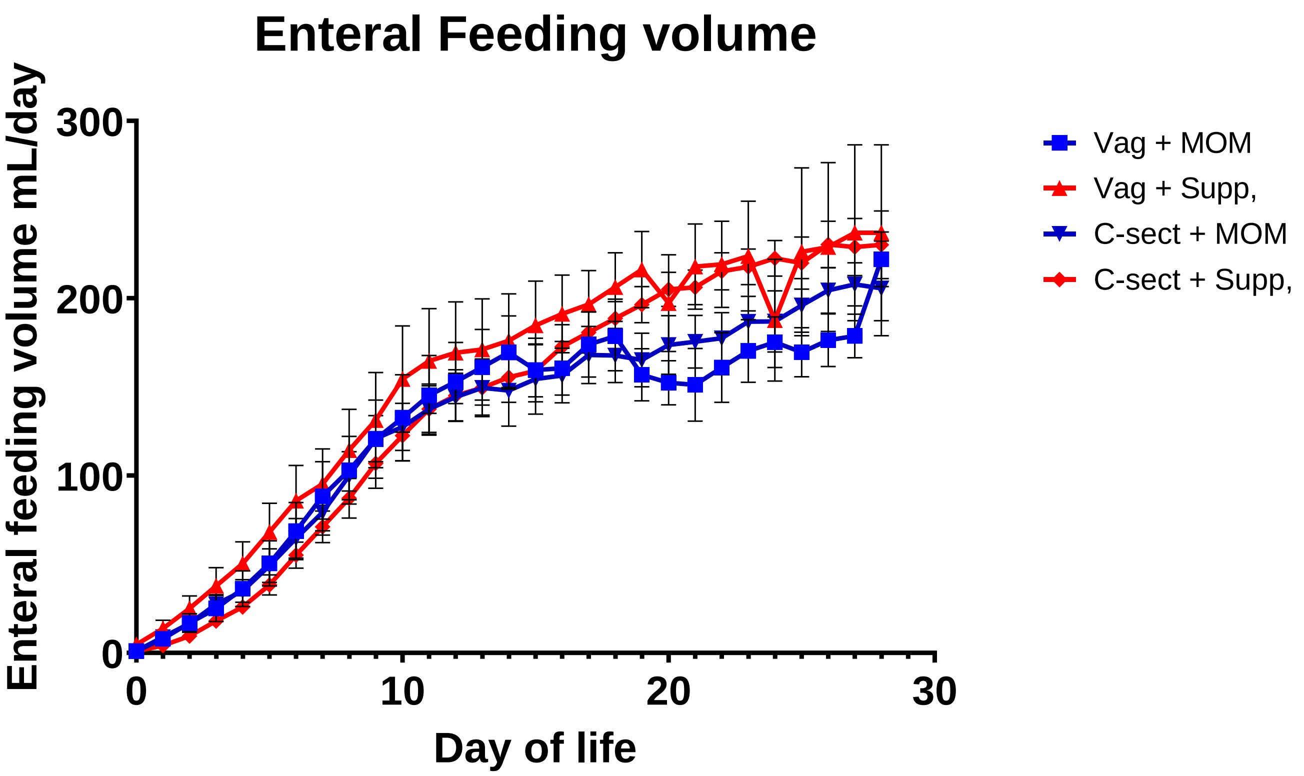

Supplementary figure 4 – Antibiotics regimen

A

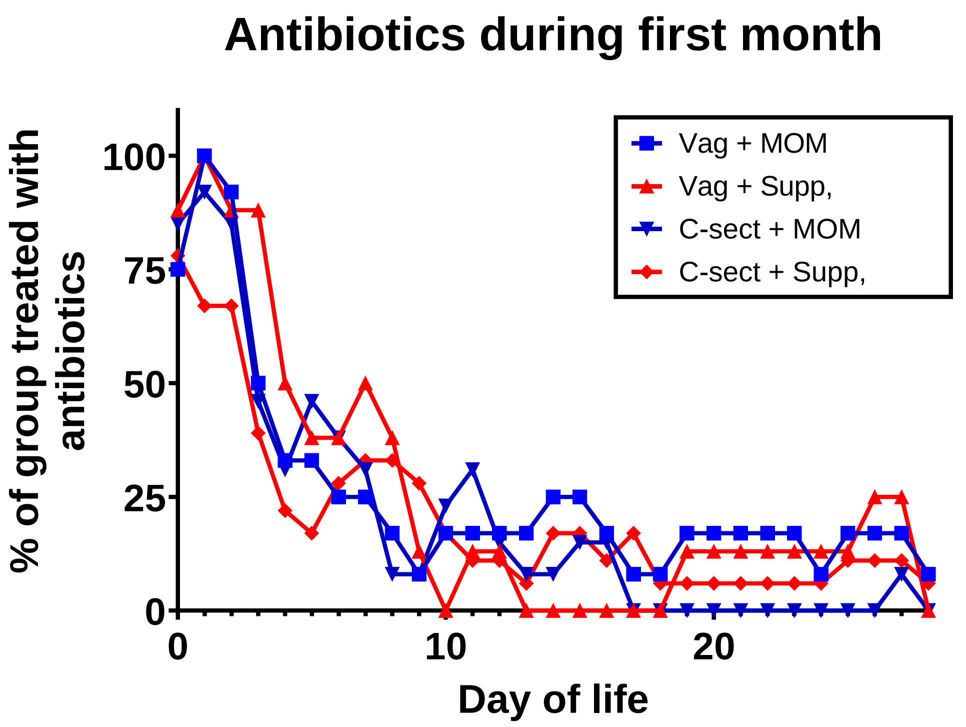

B

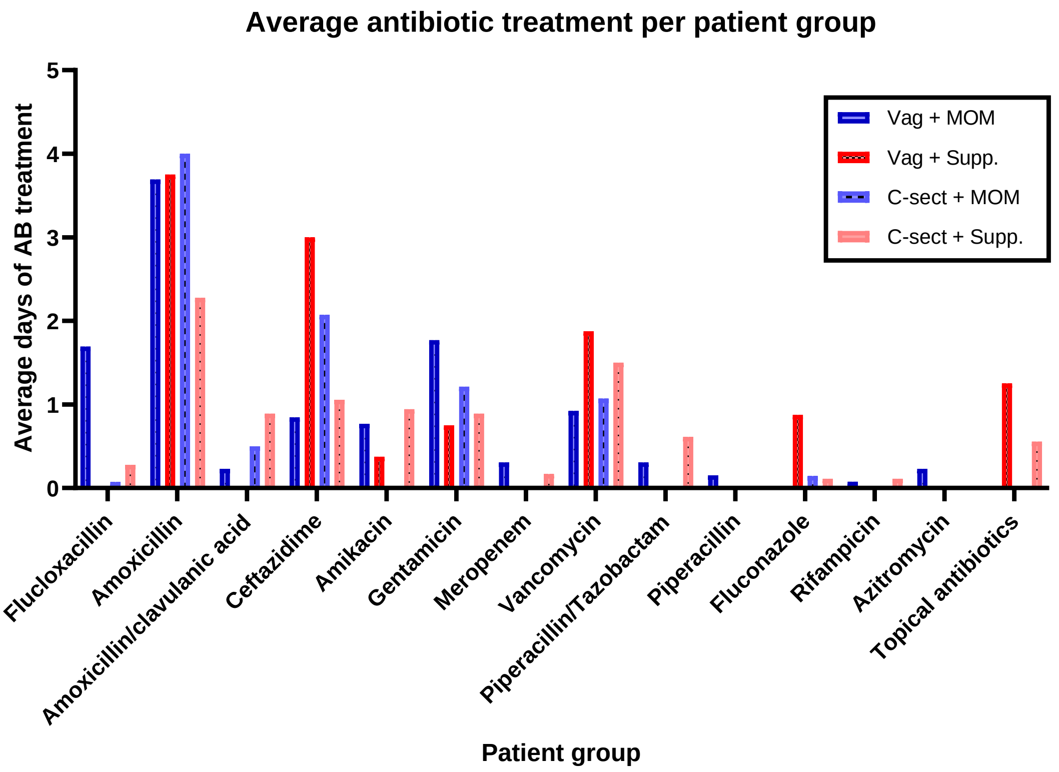

Supplementary figure 5 – Beta diversity

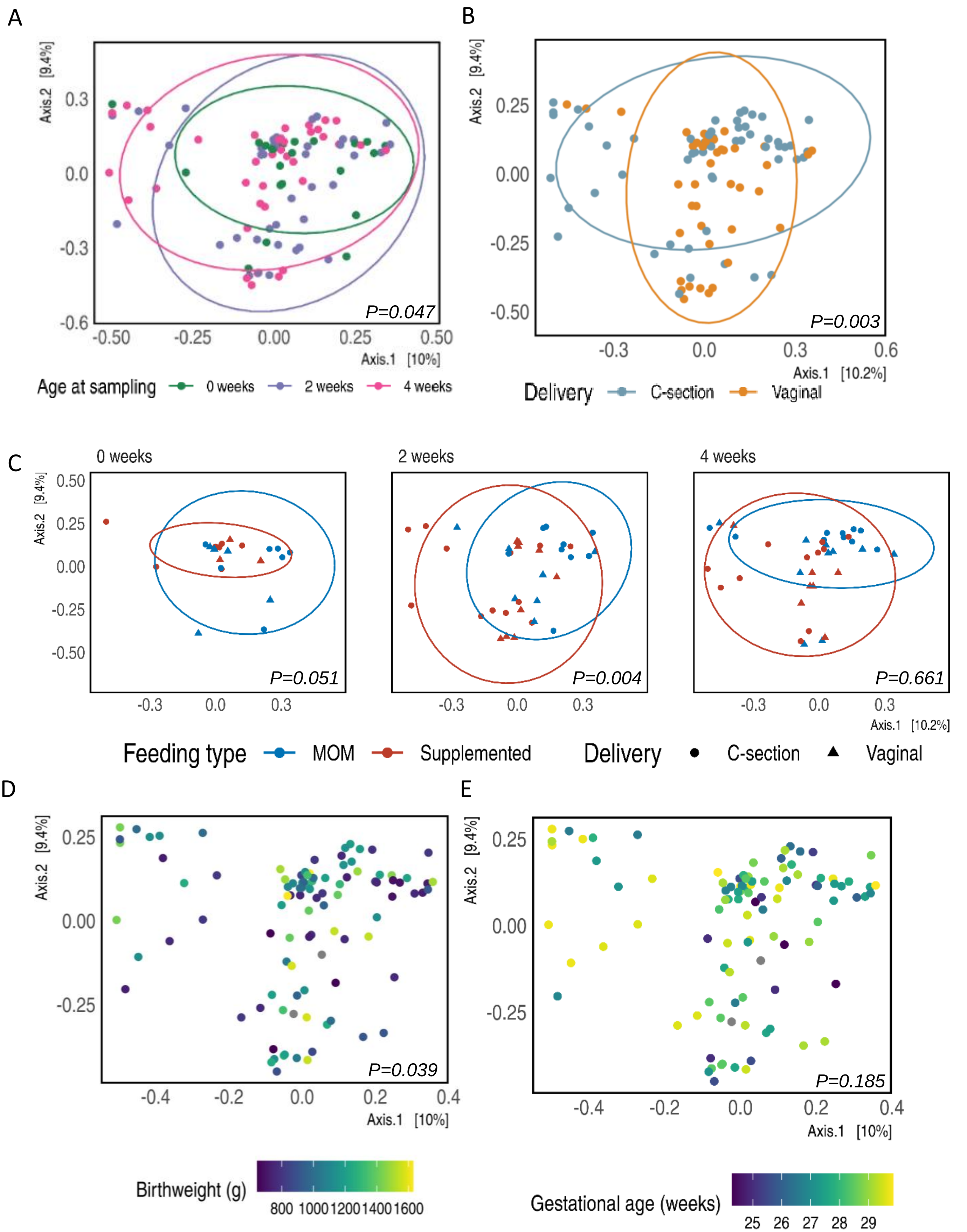

Supplementary figure 6 – Alpha diversity

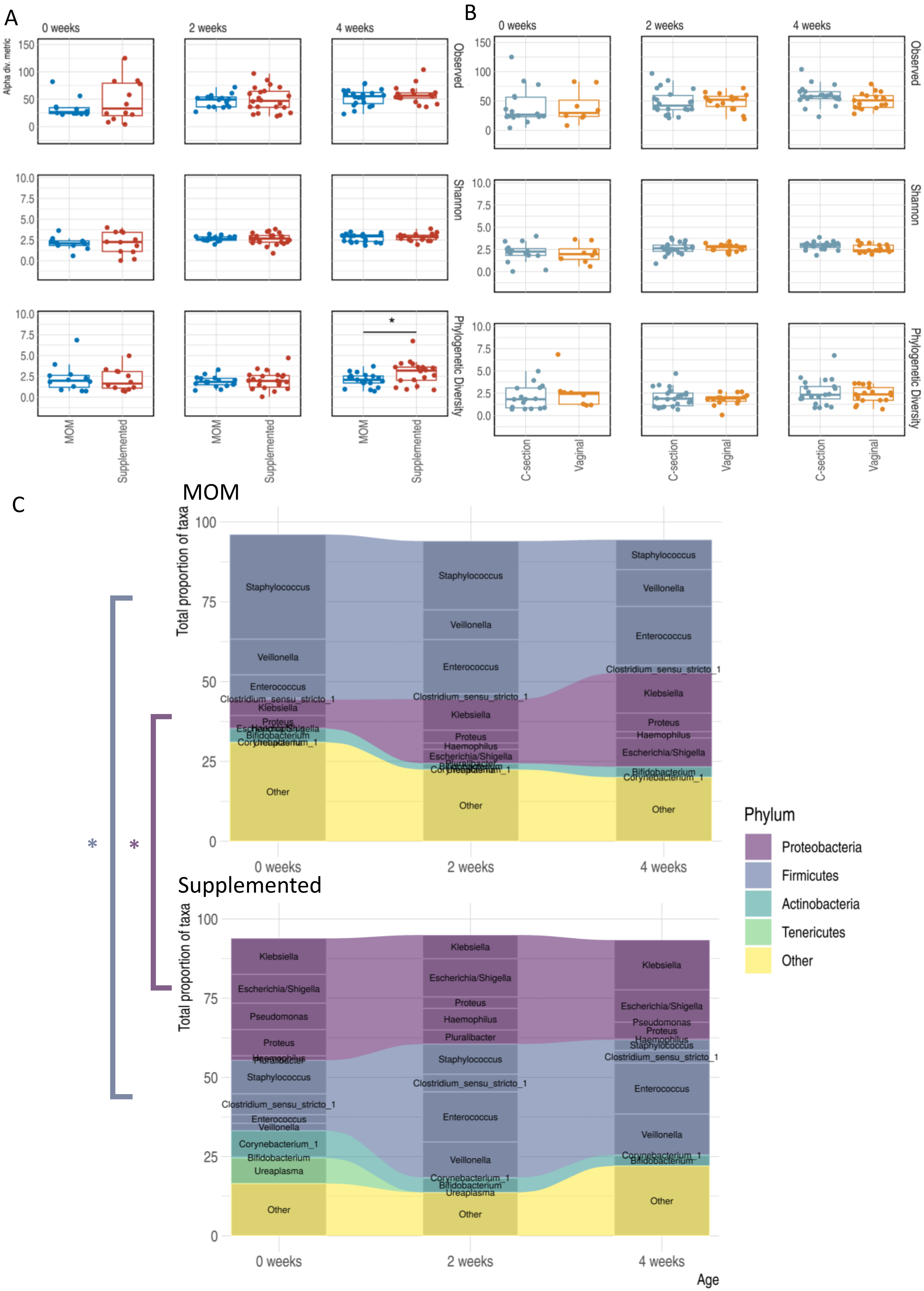

Supplementary figure 7 – Analysis of the metabolomics and baseline characteristics

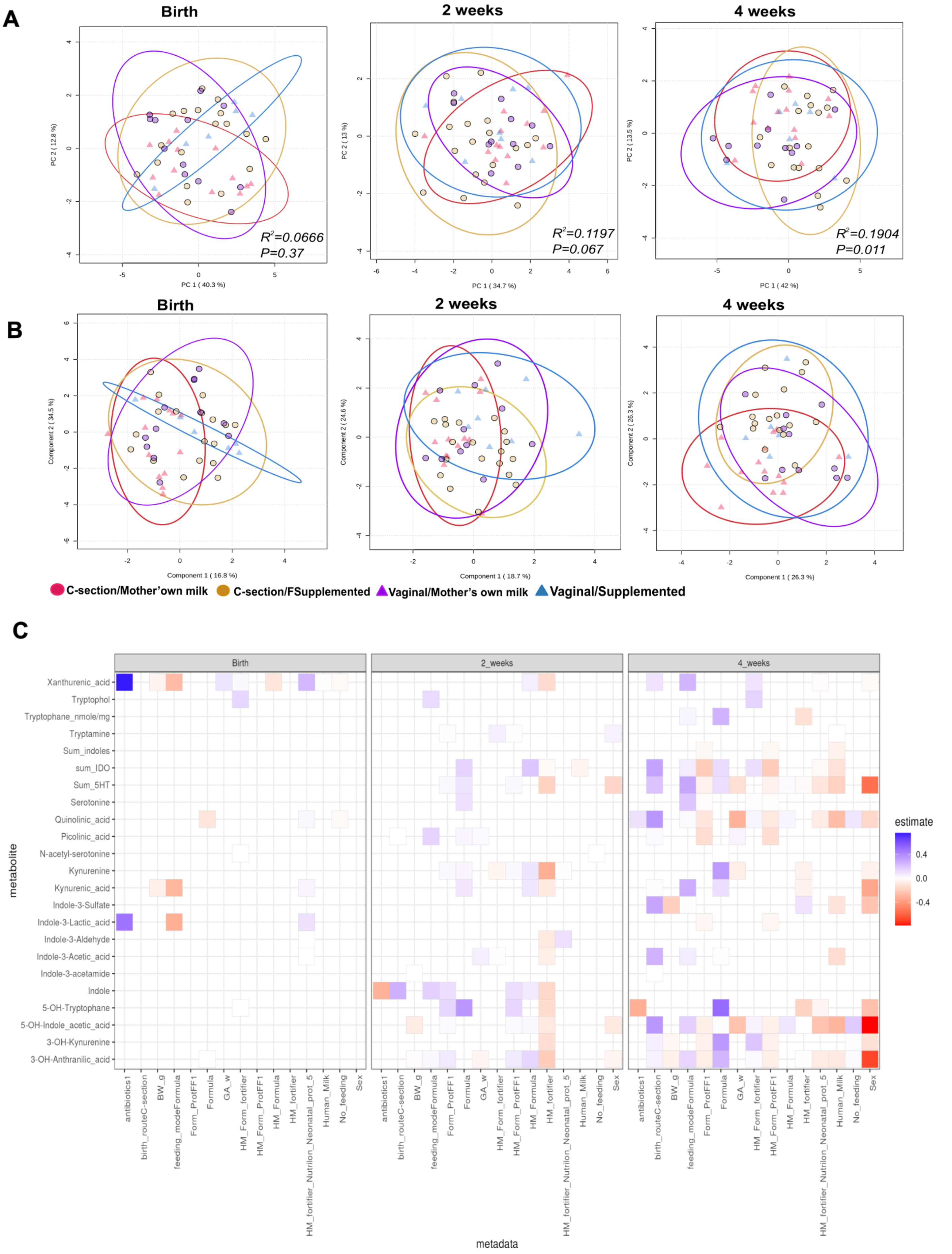

Supplementary figure 8 – Analysis of the remaining tryptophan metabolites

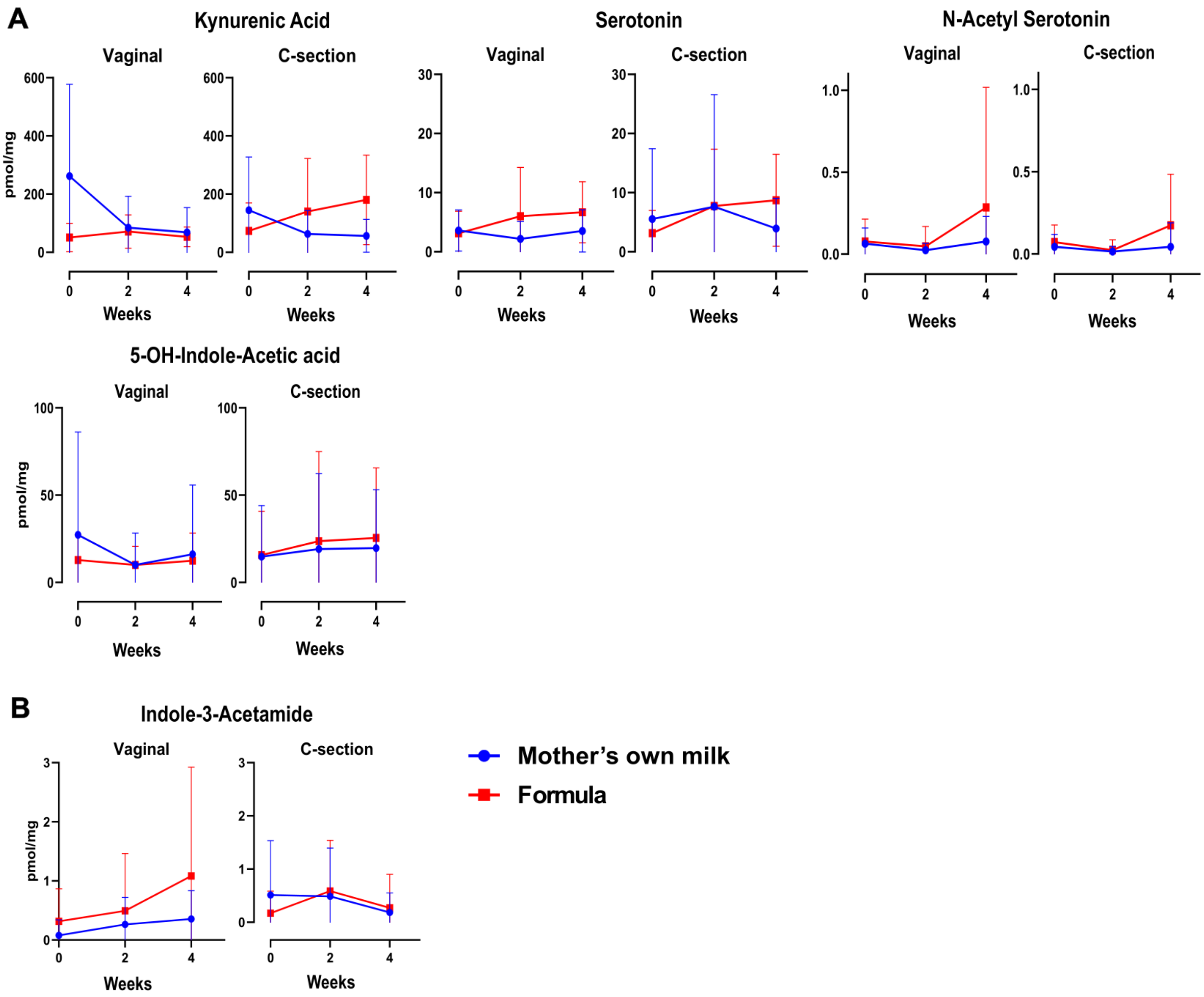

Supplementary figure 9 – Microbial-Trp associations

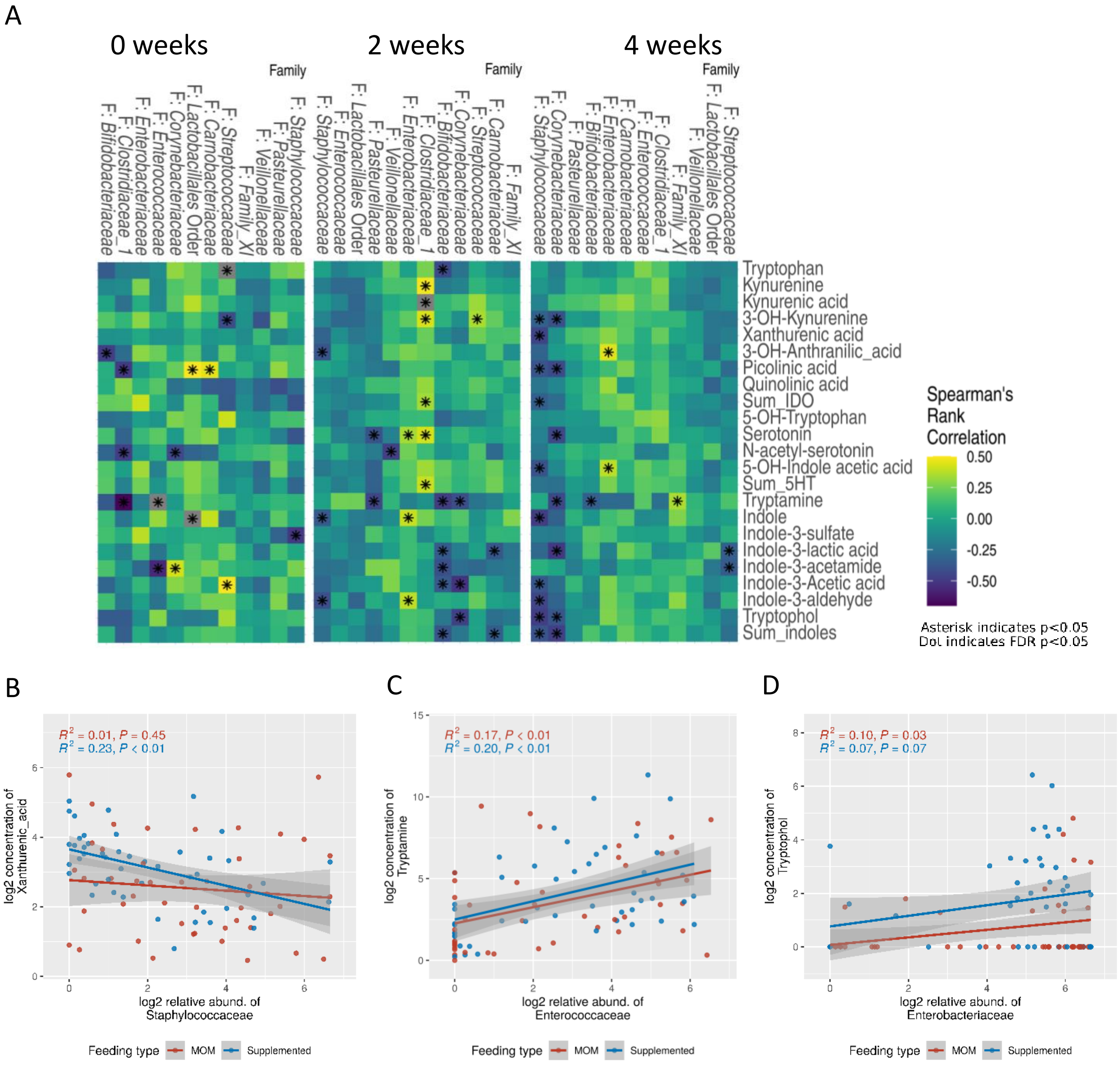

Supplementary Table1 – Daily dietary intake per group relative to total days of follow up

**Mode of birth**	*Vaginal*	*Vaginal*	*C-section*	*C-section*
**Feeding Group**	*Mothers' own milk*	*Supplemented*	*Mothers' own milk*	*Supplemented*
No enteral feeding (% of total follow up)	3.65% (SD 3.27)	0.88% (SD 1.63)	2.723% (SD 2.58)	4.11% (SD 2.77)
Exclusively MOM (% of total follow up)	37.39% (SD 18.38)	17.37% (SD 15.51)	34.39% (SD 23.24)	19.47% (SD 15.35)
Exclusively Formula (% of total follow up)	0.28% (SD 1.00)	18.87% (SD 32.45)	2.09% (SD 2.94)	22.94% (SD 33.01)
MOM + Formula (% of total follow up)	2.38% (SD 3.69)	32.54% (SD 33.20)	5.82% (SD 4.97)	19.60% (SD 17.25)
MOM + fortifier (% of total follow up)	51.30% (SD 18.10)	19.35% (SD 23.59)	54.96% (SD 24.39)	11.23% (SD 16.44
MOM + Form + fortifier (% of total follow up)	0.68% (SD 1.62)	10.50% (SD 13.88)	0% (SD 0)	21.11% (SD 18.72)
MOM + fortifier+ Nutrilon Neonatal prot. 0.5% (% of total follow up)	4.31% (SD 14.93)	0.5% (SD 1.41)	0% (SD 0)	0% (SD 0)
Form + ProtFF1% (% of total follow up)	0% (SD 0)	0% (SD 0)	0% (SD 0)	0.57% (SD 2.44)
MOM +Form+ ProtFF1% (% of total follow up)	0% (SD 0)	0% (SD 0)	0% (SD 0)	0.96% (SD 4.06)

Supplementary Table 2: overview of microbial associations to Tryptophan metabolites

S = significant Spearman LM = significant in GLM	**Human Milk**		**Formula**	
	**Positive association**	**Negative association**	**Positive association**	**Negative association**
**Tryptophan**	*S: Pasteurellaceae*<br>*LM: Streptococcaceae*		*LM: Stahpylococcaceae*	
**Kynurenine**	*S: Pasteurellaceae*			
**3-OH-Kynurenine**			*LM: Clostridiaceae_1*	*LM: Pasteurellaceae*
**3-OH-Anthranilic_acid**	***S/LM: Enterobacteriacea***		***S/LM: Enterobacteriacea***	*LM: Staphylococcaceae*
**5-OH-Tryptophane**				
**Serotonine**			*S: Clostridiaceae_1*	***S/LM: Pasteurellaceae***
**Tryptamine**	***S/LM: Enterococcaceae***	*S: Bifidobacteriaceae*	***S/LM: Enterococcaceae***<br>*LM: Family_XI Clostridia*	*S: Bifidobacteriaceae*<br>***S/LM: Corynebacteriaceae***
**Indole**			*LM: Enterobacteriaceae*<br>***S/LM: family_XI Clostridia***	***S/LM: Staphylococcaceae***
**Indole-3-acetamide**	***S/LM: Veillonellaceae***	*S: Family_XI Clostridia*		*S: Bifidobacteriaceae*
**Indole-3-Acetic_acid**		*S: Family_XI Clostridia*	*LM: Enterococcaceae*<br>***S/LM: Veillonellaceae***	*S: Bifidobacteriaceae*
**Indole-3-Lactic_acid**	*LM: Bifidobacteriaceae*		*LM: Bifidobacteriaceae*<br>*LM: Enterococcaceae*<br>*LM: Veillonellaceae*	*S: Staphylococcaceae*
**Kynurenic_acid**				*LM: Pasteurellaceae*
**Xanthurenic_acid**		*LM: Pasteurellaceae*	*LM: Bifidobacteriaceae*	***S/LM: Staphylococcaceae***
**Picolinic_acid**				
**Quinolinic_acid**				
**N-acetyl-serotonine**				*S: Staphylococcaceae*
**5-OH-Indole_acetic_acid**				*LM: Pasteurellaceae*<br>***S/LM: Staphylococcaceae***
**Tryptophol**	***S/LM: Enterobacteriaceae***		*LM: Enterococcaceae*<br>*LM: Family_XI Clostridia*	*S: Bifidobacteriaceae*
**Indole-3-Sulfate**	*LM: Enterobacteriaceae*	*LM: Staphylococcaceae*		***S/LM: Staphylococcaceae***
**Indole-3-Aldehyde**	***S/LM: Enterobacteriacea***	*LM: Staphylococcaceae*	*LM: Enterobacteriaceae*<br>*LM: Enterococcaceae*<br>*LM: Family_XI Clostridia*	*S: Staphylococcaceae*

Blue = associated to Human Milk group
Red = associated to Formula group
Underscore = associated to both groups
